# Supplementary material for: HIVprotI: an integrated web based platform for prediction and design of HIV proteins inhibitors
Source: J Cheminform. 2018 Mar 9;10:12. doi: 10.1186/s13321-018-0266-y (PMC5845081; doi:10.1186/s13321-018-0266-y)
Supplement: Supplementary file 2 — Additional file 2. Source code of HIVProtI web server. [file 13321_2018_266_MOESM2_ESM.zip › HIVprotI_Source-code/hivprotI/weka-3-6-6/doc/weka/core/matrix/FloatingPointFormat.html]

FloatingPointFormat


---


|  |  |  |
| --- | --- | --- |
| **Overview** \| **Package** \| **Class** \| **Tree** \| **Deprecated** \| **Index** \| **Help** \| **Weka's home** | |  |
| **PREV CLASS**   **NEXT CLASS** | **FRAMES**    **NO FRAMES**     **All Classes** |  |
| SUMMARY: NESTED \| FIELD \| CONSTR \| METHOD | DETAIL: FIELD \| CONSTR \| METHOD |  |


---


## weka.core.matrix Class FloatingPointFormat

```
java.lang.Object
  java.text.Format
      java.text.NumberFormat
          java.text.DecimalFormat
              weka.core.matrix.FloatingPointFormat
```

**All Implemented Interfaces:**: java.io.Serializable, java.lang.Cloneable, RevisionHandler

---

``` public class FloatingPointFormat extends java.text.DecimalFormat implements RevisionHandler ```

Class for the format of floating point numbers

**Version:**
:   $Revision: 1.4 $

**Author:**
:   Yong Wang

**See Also:**: Serialized Form

---

**Nested Class Summary**

**Nested classes/interfaces inherited from class java.text.NumberFormat**

`java.text.NumberFormat.Field`


**Field Summary**

**Fields inherited from class java.text.NumberFormat**

`FRACTION_FIELD, INTEGER_FIELD`


**Constructor Summary**

`FloatingPointFormat()`
  
          Default constructor

`FloatingPointFormat(int digits)`

`FloatingPointFormat(int w,
int d)`

`FloatingPointFormat(int w,
int d,
boolean trailingZeros)`


| **Method Summary** | |
| --- | --- |
| `java.lang.StringBuffer` | `format(double number, java.lang.StringBuffer toAppendTo, java.text.FieldPosition pos)` |
| `java.lang.String` | `getRevision()`             Returns the revision string. |
| `static java.lang.String` | `pattern(int w, int d)` |
| `int` | `width()` |

**Methods inherited from class java.text.DecimalFormat**

`applyLocalizedPattern, applyPattern, clone, equals, format, format, formatToCharacterIterator, getCurrency, getDecimalFormatSymbols, getGroupingSize, getMaximumFractionDigits, getMaximumIntegerDigits, getMinimumFractionDigits, getMinimumIntegerDigits, getMultiplier, getNegativePrefix, getNegativeSuffix, getPositivePrefix, getPositiveSuffix, getRoundingMode, hashCode, isDecimalSeparatorAlwaysShown, isParseBigDecimal, parse, setCurrency, setDecimalFormatSymbols, setDecimalSeparatorAlwaysShown, setGroupingSize, setMaximumFractionDigits, setMaximumIntegerDigits, setMinimumFractionDigits, setMinimumIntegerDigits, setMultiplier, setNegativePrefix, setNegativeSuffix, setParseBigDecimal, setPositivePrefix, setPositiveSuffix, setRoundingMode, toLocalizedPattern, toPattern`

**Methods inherited from class java.text.NumberFormat**

`format, format, getAvailableLocales, getCurrencyInstance, getCurrencyInstance, getInstance, getInstance, getIntegerInstance, getIntegerInstance, getNumberInstance, getNumberInstance, getPercentInstance, getPercentInstance, isGroupingUsed, isParseIntegerOnly, parse, parseObject, setGroupingUsed, setParseIntegerOnly`

**Methods inherited from class java.text.Format**

`format, parseObject`

**Methods inherited from class java.lang.Object**

`getClass, notify, notifyAll, toString, wait, wait, wait`

**Constructor Detail**

### FloatingPointFormat

```
public FloatingPointFormat()
```

:   Default constructor

---


### FloatingPointFormat

```
public FloatingPointFormat(int digits)
```

---


### FloatingPointFormat

```
public FloatingPointFormat(int w,
                           int d)
```

---


### FloatingPointFormat

```
public FloatingPointFormat(int w,
                           int d,
                           boolean trailingZeros)
```


**Method Detail**

### format

```
public java.lang.StringBuffer format(double number,
                                     java.lang.StringBuffer toAppendTo,
                                     java.text.FieldPosition pos)
```

:   **Overrides:**: `format` in class `java.text.DecimalFormat`

---


### pattern

```
public static java.lang.String pattern(int w,
                                       int d)
```

---


### width

```
public int width()
```

---


### getRevision

```
public java.lang.String getRevision()
```

:   Returns the revision string.

    :   **Specified by:**: `getRevision` in interface `RevisionHandler`
    :   **Returns:**: the revision


---


|  |  |  |
| --- | --- | --- |
| **Overview** \| **Package** \| **Class** \| **Tree** \| **Deprecated** \| **Index** \| **Help** \| **Weka's home** | |  |
| **PREV CLASS**   **NEXT CLASS** | **FRAMES**    **NO FRAMES**     **All Classes** |  |
| SUMMARY: NESTED \| FIELD \| CONSTR \| METHOD | DETAIL: FIELD \| CONSTR \| METHOD |  |


---
